# Stem cells, cell therapies, and bioengineering in lung biology and diseases 2023

**DOI:** 10.1152/ajplung.00052.2024

**Published:** 2024-05-21

**Authors:** Robert E. Hynds, Chelsea M. Magin, Laertis Ikonomou, Yael Aschner, Michael F. Beers, Janette K. Burgess, Rebecca L. Heise, Patrick S. Hume, Anna D. Krasnodembskaya, Shirley H. J Mei, Alexander V. Misharin, Jin-Ah Park, Susan D. Reynolds, Daniel J. Tschumperlin, Alicia E. Tanneberger, Sriram Vaidyanathan, Christopher M. Waters, Patricia J. Zettler, Daniel J. Weiss, Amy L. Ryan

**Affiliations:** ^1^Epithelial Cell Biology in ENT Research Group, Developmental Biology and Cancer Department, UCL Great Ormond Street Institute of Child Health, University College London, London, United Kingdom; ^2^Division of Pulmonary Sciences and Critical Care Medicine, Department of Medicine, University of Colorado Anschutz Medical Campus, Aurora, Colorado, United States; ^3^Department of Bioengineering, University of Colorado Anschutz Medical Campus, Aurora, Colorado, United States; ^4^Department of Pediatrics, University of Colorado Anschutz Medical Campus, Aurora, Colorado, United States; ^5^Department of Oral Biology, University at Buffalo, State University of New York, Buffalo, New York, United States; ^6^Division of Pulmonary, Critical Care and Sleep Medicine, Department of Medicine, University at Buffalo, State University of New York, Buffalo, New York, United States; ^7^Pulmonary, Allergy, and Critical Care Division, Department of Medicine and PENN-CHOP Lung Biology Institute, Perelman School of Medicine at the University of Pennsylvania, Philadelphia, Pennsylvania, United States; ^8^Department of Pathology and Medical Biology, University Medical Center Groningen, Groningen, The Netherlands; ^9^Department of Biomedical Engineering, Virginia Commonwealth University, Richmond, Virginia, United States; ^10^Division of Pulmonary, Critical Care, and Sleep Medicine, Department of Medicine, National Jewish Health, Denver, Colorado, United States; ^11^Wellcome-Wolfson Institute for Experimental Medicine, School of Medicine, Dentistry and Biomedical Sciences, Queen’s University Belfast, Belfast, United Kingdom; ^12^Regenerative Medicine Program, Ottawa Hospital Research Institute, Ottawa, Ontario, Canada; ^13^Division of Pulmonary and Critical Care Medicine, Feinberg School of Medicine, Northwestern University, Chicago, Illinois, United States; ^14^Department of Environmental Health, Harvard T.H. Chan School of Public Health, Harvard University, Boston, Massachusetts, United States; ^15^Center for Perinatal Research, Abigail Wexner Research Institute at Nationwide Children's Hospital, Columbus, Ohio, United States; ^16^Department of Physiology and Biomedical Engineering, Mayo Clinic College of Medicine and Science, Rochester, Minnesota, United States; ^17^Center for Gene Therapy, Abigail Wexner Research Institute at Nationwide Children’s Hospital, Columbus, Ohio, United States; ^18^Department of Physiology and Saha Cardiovascular Research Center, University of Kentucky, Lexington, Kentucky, United States; ^19^Moritz College of Law, Drug Enforcement and Policy Center, The James Comprehensive Cancer Center, The Ohio State University, Columbus, Ohio, United States; ^20^Department of Medicine, University of Vermont, Burlington, Vermont, United States; ^21^Department of Anatomy and Cell Biology, Carver College of Medicine, University of Iowa, Iowa City, Iowa, United States

**Keywords:** airway epithelium, alveolar epithelium, extracellular matrix, immune cells, lung regeneration

## Abstract

Repair and regeneration of a diseased lung using stem cells or bioengineered tissues is an exciting therapeutic approach for a variety of lung diseases and critical illnesses. Over the past decade, increasing evidence from preclinical models suggests that mesenchymal stromal cells, which are not normally resident in the lung, can be used to modulate immune responses after injury, but there have been challenges in translating these promising findings to the clinic. In parallel, there has been a surge in bioengineering studies investigating the use of artificial and acellular lung matrices as scaffolds for three-dimensional lung or airway regeneration, with some recent attempts of transplantation in large animal models. The combination of these studies with those involving stem cells, induced pluripotent stem cell derivatives, and/or cell therapies is a promising and rapidly developing research area. These studies have been further paralleled by significant increases in our understanding of the molecular and cellular events by which endogenous lung stem and/or progenitor cells arise during lung development and participate in normal and pathological remodeling after lung injury. For the 2023 Stem Cells, Cell Therapies, and Bioengineering in Lung Biology and Diseases Conference, scientific symposia were chosen to reflect the most cutting-edge advances in these fields. Sessions focused on the integration of “omics” technologies with function, the influence of immune cells on regeneration, and the role of the extracellular matrix in regeneration. The necessity for basic science studies to enhance fundamental understanding of lung regeneration and to design innovative translational studies was reinforced throughout the conference.

## Graphical abstract

**Figure:**
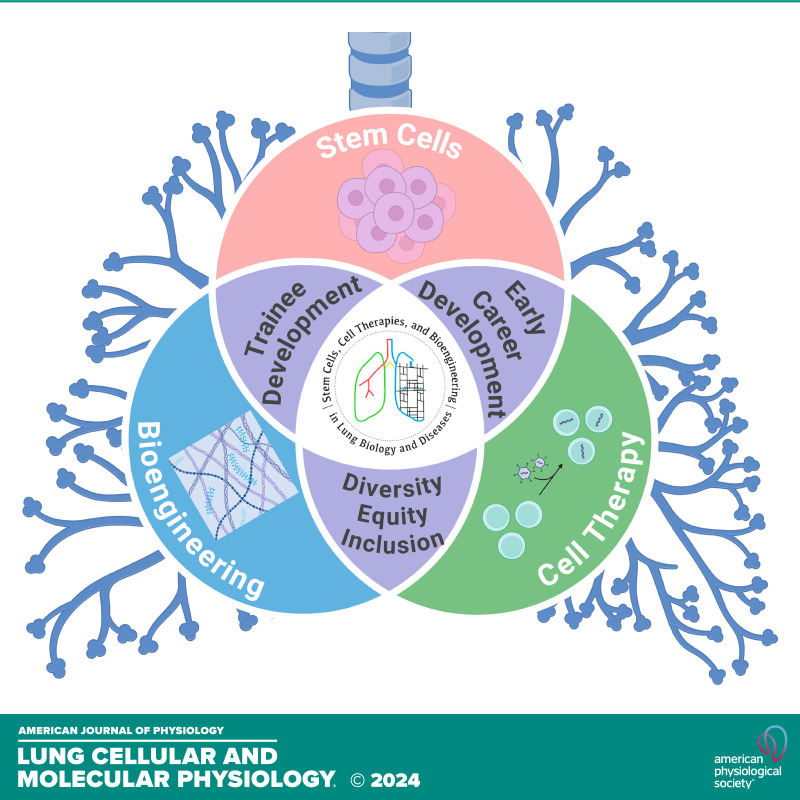

## INTRODUCTION

There continues to be a rapid explosion and increasing interest in regenerative medicine approaches for lung diseases and critical illnesses. Indeed, the application of new and emerging technologies to lung stem cell biology and regeneration has led to exciting advances in the field in the 2 years since the last conference. The necessity for basic science studies to enhance fundamental understanding of lung regeneration and to design innovative translational studies was a core theme addressed at the 10th “Stem Cells, Cell Therapies, and Bioengineering in Lung Biology and Diseases” Conference. Essential investigation of the mechanisms that mobilize endogenous cells is essential for developing approaches for repairing damaged airway and alveolar epithelium. Furthermore, understanding the complexity of the regulation of lung stem and progenitor cells by their direct, in vivo, niche is vital to the success of any approach that aims to restore homeostasis in a diseased lung. For the 2023 conference, scientific symposia were chosen to reflect the most cutting-edge advances in the field and included sessions focusing on the integration of “omics” technologies with function, the influence of immune cells on regeneration, and understanding the role of the extracellular matrix (ECM) in regeneration.

The 2023 10th biennial conference (1–8) saw continued leadership from Dr. Amy Ryan (Chair and Associate Professor, University of Iowa), with Dr. Chelsea Magin (Assistant Professor, University of Colorado) as the Vice-Chair. Dr. Daniel Weiss (Professor, University of Vermont), cofounder of the conference in 2005, remained on the committee as Emeritus Chair. The conference was organized by a planning committee broadly representing researchers from the United States and Europe and was supported by a junior planning committee led by Dr. Robert Hynds (Senior Research Fellow, University College London, UK), who performed a mentored review of abstracts and organized all poster presentation sessions at the conference. This committee comprised trainees and junior faculty from five different countries with 50% female and 50% other underrepresented groups.

Since the inaugural meeting in 2005, the field has continued to move at a rapid pace with many recent advances. The field has witnessed substantial improvements in the differentiation and maturation of induced pluripotent stem cell (iPSC)-derived airway cells and expanded to investigate a wider variety of cells that might participate in lung repair and regeneration. There has been a significant shift to focus on the regulation of stem cells by their surrounding niche. Increased evidence of the potential role of cell-derived products, such as extracellular vesicles, in orchestrating repair and regeneration via immunomodulation of the lung is being generated. Furthermore, our understanding of the impact of aging on the biology of endogenous lung progenitor cells that might participate in repair and remodeling after lung injury and that might also function as tumor-initiating cells in the lung has improved alongside technological advances in identifying and characterizing putative populations of human airway progenitor cells. Finally, rapidly developing bioengineering approaches, including the development of more complex three-dimensional (3-D) models and the generation of precisely engineered biomaterials, are allowing the field to move toward better and more physiological disease models. These recent advances were the focus of the scientific sessions at the conference.

## BROADER IMPACT ON THE RESEARCH COMMUNITY

### Geographic Representation

**Figure 1.**
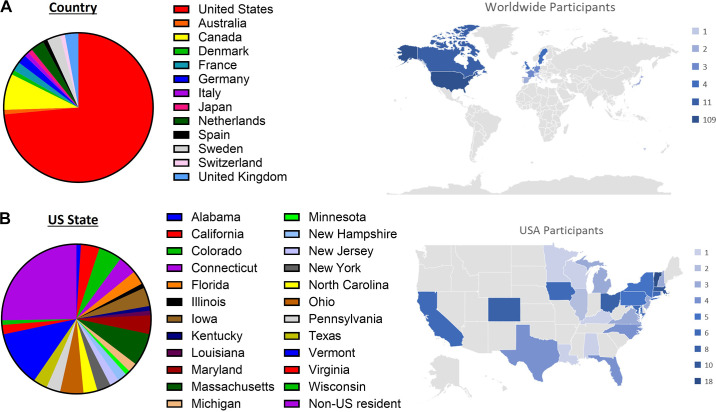
Countries and states represented at the 2023 conference. Pie charts depict geographic location of attendees by country (*A*) and US state (*B*).

In 2023, the conference attracted a total of 143 participants. There were 43 oral presentations, and trainees presented 46 posters and 7 oral presentations. Significant global and geographical outreach was highlighted, with 13 countries and 23 US states represented among attendees (Fig. 1).

### Inclusion of Women, Underrepresented Groups, and Persons with Disabilities

**Figure 2.**
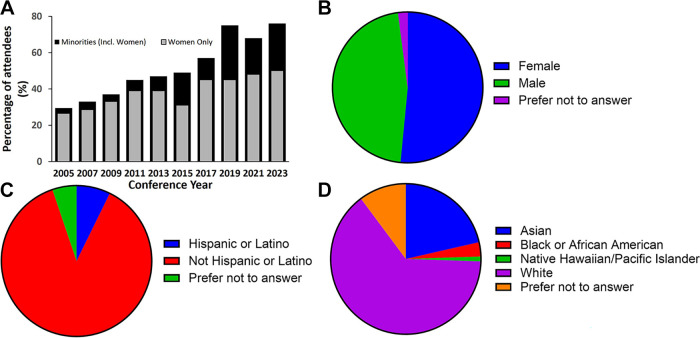
Representation of sex, race, and ethnicity at the 2023 conference. *A*: bar chart shows change in inclusion of underrepresented groups (minorities and women) since the inception of the conference. Pie charts show attendance by sex (*B*), ethnicity (*C*), and race (*D*).

Underrepresented groups, including women, people with disabilities, and those defined as underrepresented in science and medicine by the National Institutes of Health (NIH), were included in planning the conference [Chairs, Planning, Women’s/Diversity, and the Junior Planning Committee (JPC)] and as investigator and trainee participants. Although these proportions partially reflect the demographics of relevant scientific researchers, a continued effort was made to increase diversity and inclusion, particularly among trainees. The 2023 conference had 50% female participation (Fig. 2, *A* and *B*) and 10% of participants identifying as Hispanic/Latino/Black or African-American (Fig. 2*C*).

### Emphasis on Trainee and Junior Investigator Career Development

Importantly, a strong emphasis of the conference has is the active participation of junior faculty and trainees. The 2023 conference saw significant growth in trainee participation and in international and underrepresented groups (Fig. 2, *C*–*D*). To further emphasize trainee development, highly successful features introduced in the last four meetings (2015–2021) included presentations and interactive panel discussions on career development, a mentoring lunch pairing trainees with selected senior investigators, a women’s/diversity session and networking panel discussion, and seven travel awardees for Outstanding Abstracts who were given the opportunity to present their data in the scientific sessions. Specifically, two of these Outstanding Abstract awards were designated for women, underrepresented groups, or persons with disabilities.

## PRECONFERENCE SKILL DEVELOPMENT HANDS-ON WORKSHOP

The meeting began with a full day hands-on session for training in relevant experimental approaches in the study of lung diseases and regenerative medicine. This workshop provided an interactive platform to discuss the application of cutting-edge technologies to rapidly evolving areas of lung repair and regeneration (9). In 2023, new hands-on topics included precision-cut lung slices (PCLS), organoids and hydrogels and biomechanical regulation of lung function, all at the forefront of the field. In addition to parallel morning sessions, a focused bioinformatics workshop was the feature of the afternoon in response to prior feedback on skills needed in the laboratory. As a new feature in 2023, a program including eight local high school science, technology, engineering and mathematics (STEM) students in the workshop was implemented. The relatively small size of the meeting, coupled with the diversity of scientific expertise in stem cells, lung biology, and bioengineering, provided an ideal opportunity to foster interactions and collaborations between investigators and trainees.

## SCIENTIFIC FOCUS ON STEM CELLS, CELL THERAPY, AND BIOENGINEERING IN LUNG BIOLOGY AND DISEASE

Beginning in 2005, this meeting was the first and remains the only ongoing meeting globally to specifically focus on the role of stem cells, cell therapies, and bioengineering in lung biology and disease. The meeting has repeatedly attracted leading national and international experts and been marked by spirited and vigorous discussion and debate on key issues impacting the field. The 2023 conference was no exception. Conducted over a 4-day period, the meeting featured presentations from stem cell and lung biology experts, as well as experts in other relevant disciplines, followed by research presentations from promising junior investigators. Each session included ample time for discussion and debate.

### Session I: Engineering New Models of Lung Disease and Repair

The first session opened the conference by highlighting technological innovations that are driving research and development to create a potentially unlimited supply of 3-D-printed, functional lungs. Dr. Keri Dame (Senior Scientist at United Therapeutics) shared progress in 3-D modeling, bioink design, high-resolution 3-D printing technologies, and autologous recellularization approaches. Under good manufacturing practices (GMPs), preproduction 3-D-printed lung models feature resolution down to 11.3 μm, as well as cellular attachment, proliferation, and maturation in long-term culture up to 100 days. This provided an exciting advance in the translation of these technologies from ideas in the laboratory (10) to preproduction in industry. Following this, Dr. Jeremy Hirota (Associate Professor, McMaster University) introduced the concept of “kaizen” or continuous improvement for preclinical models of lung disease. The talk described the process of adding physiological signals, including stretch and air-liquid interfaces to models of lung disease and exposure. The cell and tissue (CaT) stretcher highlighted in this presentation included *1*) programmable breathing patterns, *2*) scalability to support high-throughput screening, and *3*) modularity for submerged cell culture, air-liquid interface culture, organs-on-chips, and/or tissue samples (11). A core concept of rebuilding the microphysical environment or niche as it exists in the human lung prevailed throughout this session, with Dr. Claudia Loebel (Assistant Professor, University of Michigan) presenting a facile approach for culturing alveolospheres in a microstructured hydrogel. This hydrogel was made of hyaluronic acid, creating a Matrigel-free culture of iPSC-derived alveolar type 2 cells (iAT2s) (12). The system is tunable and can be used to examine the role of mechanotransduction signaling to push cellular differentiation. This innovative technology will likely form the basis for many future studies of AT2s and related mechanobiology.

Dr. Xi (Charlie) Ren (Associate Professor, Carnegie Mellon University) presented work on fabricating and quantifying rotational speed in an apical-out airway epithelial cell organoid (13, 14). They showed that rotational speed of these organoids can be used as a quantitative measure of ciliary health and proposed the utility of these organoids to investigate environmental exposures on airway epithelial function. The final presentation of the session was from outstanding abstract awardee, Dr. Sinem Koc-Gunel (Physician-Scientist at the Universität Frankfurt am Main, Germany). A multicellular spheroid model that mimics lymphangioleiomyomatosis nodules was presented. She used these organoids to test the effects of sorafenib on inhibiting cellular invasion and presented spatial transcriptomics data to point toward the mechanism of action of the nodular formation (15). This session reinforced the utility of physiologically relevant and human-engineered models in accelerating our ability to understand both lung disease pathogenesis and injury repair mechanisms.

### Session II: Lung Repair and Regeneration: Integrating Omics with Function

Four speakers, each representing different domains of omics technology and a variety of aspects of lung disease, were featured in this session. By combining cutting-edge intravital microscopy, Dr. Jamie Hook (Assistant Professor at the Icahn School of Medicine at Mount Sinai) investigated the mechanisms of alveolar barrier repair after *Staphylococcus aureus* (SA) infection (16, 17). Live imaging of SA-infected lungs showed that epithelial cells rapidly recovered barrier function after an initial injury. Exciting new data were presented, demonstrating a role for Notch signaling, previously shown to promote barrier function in endothelial cells, in mediating alveolar barrier repair after SA infection. Inhibition of Notch activation delayed barrier recovery, suggesting Notch activation as a potential therapeutic target for SA-induced acute lung injury. Dr. Ramon Sun (Associate Professor, University of Florida) then demonstrated how high-resolution spatial metabolomics, a novel omics modality, can provide unexpected insights into the pathobiology of pulmonary fibrosis (18). First, Dr. Sun’s laboratory optimized resolution (down to a 5-µm spot, comparable with the size of a typical cell), sensitivity, and throughput of the matrix-assisted laser desorption/ionization (MALDI) platform for spatial metabolomics (19, 20). Then in collaboration with Dr. Christopher Waters (Professor, University of Kentucky), he demonstrated accumulation of glycogen in myofibroblasts in the lungs from patients with pulmonary fibrosis and mouse models of pulmonary fibrosis (21). These findings suggest that metabolic channeling between glycogen and N-linked glycans is critical for pulmonary fibrosis progression. Mouse models with lysosomal glycogen use deficiency displayed reduced fibrosis, providing conclusive evidence that glycogen use is required for fibrosis progression. In addition, Dr. Sun presented the spatial augmented multiomics interface (SAMI) platform, a novel computational platform for data integration, analysis, and functional annotation across different omic modalities, which could greatly aid the rapid adoption of spatial metabolomics as part of a standard analytical workflow (19).

Dr. Alexander Misharin (Associate Professor, Northwestern University) demonstrated how bulk and single-cell transcriptomics can be applied to animal models of lung diseases and samples obtained from patients with lung diseases to provide insights into the role of the immune system during injury and repair. He presented data demonstrating that profibrotic monocyte-derived alveolar macrophages play a causal role in the pathogenesis of pulmonary fibrosis by establishing forward-feeding loops with monocyte-derived alveolar macrophages. This phenomenon is conserved across the spectrum of pulmonary fibrosis in humans, from classical interstitial lung diseases (22) to fibrotic abnormalities in patients with postacute sequalae SARS-CoV-2 infection (PASC) (23). Dr. Sam Raredon (Research Group Leader, Yale University) presented niche interactions and cellular heterogeneity in extracellular signaling (NICHES), a novel computational approach for understanding and modeling cell-to-cell interactions in the normal and diseased lung using single-cell RNA-seq (scRNA-Seq) data observed in the normal lung (24). He then demonstrated how computational models derived from NICHES analysis can be applied to guide tissue engineering of the whole lung in vitro. Using decellularized rat lungs as biomimetic scaffolds and pharmacologically expanded epithelial, endothelial, and stromal cells, he then systematically modified culture media to recreate multicellular niches and interactions observed in the normal lung (25). This approach outperformed traditional empiric approaches, resulting in improved blood perfusion, cellular maturation, and greater alveolar barrier function in engineered whole lungs.

### Session III: Heterogeneity in Lung Immune Cells and Influences on Regeneration

Recent advances in techniques including scRNA-Seq have rapidly advanced our understanding of context-dependent heterogeneity in resident and recruited immune cell phenotypes in the lungs. Dr. Jason Mock (Associate Professor, University of North Carolina) and his team have described a role for increased T_reg_ cell counts in response to acute lung injury induced by intratracheal administration of lipopolysaccharide (LPS) (26). Foxp3^+^ T_reg_ cell-depleted mice exhibited reduced AT2 cell proliferation after LPS damage, indicating hindered recovery (26). Transcriptional profiling of murine T_reg_ cells during acute lung injury indicated diminished *Kdm6b*, a lysine-specific demethylase, after LPS injury (27). Interestingly, mice with *Kdm6b*-deficient T_regs_ showed decreased *Foxp3* expression and T_reg_ cell numbers. When subjected to H1N1 influenza, *Kdm6b*-deficient mice displayed increased CD3^+^ and CD8^+^ T cells, implying an exaggerated inflammatory response. He also found that patients with acute respiratory distress syndrome (ARDS) and higher T_reg_ cell proportions in bronchoalveolar lavage (BAL) fluid correlated with shorter mechanical ventilation durations (28). Therefore, T_reg_ cells may hold promise as a cell-based therapy for ARDS, including for COVID-19, where an off-the-shelf, cryopreserved, allogeneic umbilical cord blood-derived T_reg_ cell product recently demonstrated a promising safety profile in a phase I safety trial (*n* = 30 patients; NCT04468971) (29).

Dr. Thomas Conlon (Team Leader at Helmholtz Munich) switched the focus from T to B cells and presented data on the role of B cells as potential therapeutic targets in chronic obstructive pulmonary disease (COPD). They demonstrated that B cell-deficient mice are protected from airspace enlargement after cigarette smoke exposure, and that these mice displayed less lung inflammation and smoke-induced bronchus-associated lymphoid tissue (iBALT) formation (30). They identified lymphotoxin beta receptor (LTβR) as one of the key mediators of COPD pathogenesis (31). LTβR levels were elevated in mice exposed to chronic cigarette smoke and in patients with COPD. This led to the activation of the noncanonical nuclear factor kappa B (NF-κB) pathway, resulting in inflammation and tissue damage. An LTβR-Ig fusion protein inhibited LTβR signaling and iBALT formation, which resulted in Wnt/β-catenin pathway activation and enhanced alveolar epithelial progenitor cell growth in both mouse and human derived cells (31). Treating mice with LTβR-Ig protected against emphysema development over a 4-mo period of smoke exposure, indicating significant therapeutic potential (31).

Next, Dr. Claudia Jakubzick (Associate Professor, Dartmouth University) discussed the pivotal role of macrophages in lung development, homeostasis, and repair. Transcriptional profiling of human BAL samples revealed 14 airspace macrophage subclusters that can be grouped into 4 superclusters by variable *IFI27* and *APOC2* expression (32). The ratios of these clusters are consistent across individuals with or without cystic fibrosis. Their recent research centers on the functional heterogeneity of tissue-based interstitial macrophages (IMs) (33). In mice, they identified seven chemokine-expressing IM subsets in healthy lung tissue or after LPS inflammation. These chemokine signatures span various pulmonary and extrapulmonary tissue and across species (mouse and human). To investigate the role of CD206^high^ IMs, they used selective PF4/CXCL4 depletion, which resulted in the reduced influx of inflammatory lymphoid cells and suggested macrophage coordinate inflammatory responses (33). The final talk of the session was by Dr. Patrick Hume (Assistant Professor at National Jewish Health), who was an Outstanding Abstract awardee. He discussed recent work from his laboratory on human IMs, which are distributed throughout all lung tissue sublayers (34). Using tissue-specific transcriptional profiling on alveolar, pleural, vessel wall, and airway wall tissues, they defined the local IM landscape. They identified five IM subtypes with varying accumulation across lung subtissues, including a novel population marked by folate receptor β^+^/interleukin-1β^+^ in the vessel walls that may coordinate T cell responses. Overall, each of the above findings in lymphocytes and macrophages shed light on the intricate lung immune mechanisms with exciting potential for therapeutic targeting to coordinate lung repair and regeneration.

### Session IV: Careers in Stem Cells, Cell Therapies, and Lung Bioengineering

This session started with a keynote presentation entitled “Pursuing Translational Biomedical Science in an Academic Lab” by Dr. Polly Parsons (Professor, University of Vermont) and was followed by a panel discussion entitled “Women and Diversity.” Dr. Parsons used her undergraduate research experience to emphasize the benefits of a diverse knowledge base. She then provided examples of successful mentoring and how the mentor-trainee roles can be reversed as trainees become independent. The panel discussion, moderated by Dr. Chelsea Magin (Assistant Professor, University of Colorado, Denver | Anschutz), was focused on various sources of diversity, including gender, race, ethnicity, nationality, sexual orientation, disability status, and neurodiversity. The panelists started by introducing themselves and aspects of their identity that were important for them to emphasize throughout their careers. Both panelists and audience members shared numerous poignant examples of how discrimination can limit career development. Thought-provoking concepts that emerged included the idea that diversity attracts diversity and that creation of an inclusive laboratory environment requires daily effort. Individuals were challenged to be confident in themselves and in their science, to resist comparisons with others, and to challenge themselves. Allies were challenged to speak up and continue to create inclusive spaces for all groups. Career advice included the importance of selecting some, but not all, service/committee opportunities and the need to identify and use mentors, sponsors, and coaches.

### Session V: Application of Surgical Techniques to Advance Stem Cell Therapeutics

Several hurdles remain to develop translational epithelial cell and/or gene therapies. These include the development of protocols to enable cell or gene therapy delivery, consideration of optimal scaffold materials for cell engraftment, methods to monitor the performance of lung grafts, and optimization of cell culture protocols to obtain suitable cells (35). This session presented existing clinical techniques and animal models that enable us to optimize the delivery of cell and gene therapies (CGTs) and accelerate progress toward new CGTs for airway diseases.

The first two talks examined surgical and bronchoscopic approaches for the delivery of cells into various parts of the airways and scaffolds that may enable cell delivery. Work from Dr. Tendy Chiang (Assistant Professor at Nationwide Children’s Hospital) investigated the use of partially decellularized tracheal grafts as an approach to treat large-segment proximal airway defects. The partial decellularization approach removes epithelial and stromal cells but preserves tracheal cartilage, chondrocytes, and the mechanical properties of the native tissue. Within 2 wk of orthotopic transplantation, tracheal grafts are repopulated by host-derived neotissue that recapitulate the cellular diversity of the normal trachea (36). Single-cell RNA sequencing data suggested that host basal cells remain in an activated state 3 and even 6 mo after surgery. Dr. Chiang also presented their adaptation of a previously described rabbit tracheal surgical approach for long-segment defects (37, 38). He noted that rabbit airway dimensions are similar to human neonates and that the rabbit proximal airway exhibits structural and functional parallels to human. However, these similarities do not extend to the larynx, an organ for which reconstructive approaches are also needed, but other animals, such as sheep, might provide better large animal models.

Consistent with a growing interest in the rabbit model system, Dr. Do-Yeon Cho (Associate Professor, University of Alabama at Birmingham) presented work using rabbits as a model for chronic rhinosinusitis. Blocking the sinus outflow tract by insertion of a sterile sponge results in acute inflammation within the sinus mucosa and develops into a chronic inflammatory condition by 14 wk (39, 40). This model can be used for therapeutic testing (41) and Dr. Cho presented data, suggesting that the rabbit sinonasal cavity represents a viable model for cell transplantation studies using endoscopic delivery of cells and scaffolds. Given the feasibility of gene correction in airway basal cells (42–44), successful transplantation would represent a major step toward new therapies for cystic fibrosis and other monogenic lung diseases. In the discussion, some of challenges of working with rabbits were highlighted, specifically with regard to anesthesia protocols and antibody availability.

Ada Sher (Medical Student, Ohio State University) presented a comprehensive overview of methodologies for monitoring mucociliary clearance in human patients. The talk highlighted the availability of methods, including imaging of radiolabeled aerosols using single-photon emission computed tomography (SPECT), that can be used to noninvasively quantify mucociliary escalator function in both patients (45) and animal models (46).

Dr. Jessica Orr (Postdoctoral Researcher, University College London, UK) was the Outstanding Abstract awardee in this session and presented work to develop a higher throughput screening system to study regulators of airway basal cell proliferation. Following isolation and expansion of airway basal cells in coculture with mouse embryonic 3T3-J2 feeder cells (47, 48), cells were transduced with a lentiviral luciferase reporter construct and treated with compounds from two compound screening libraries, which include many US Food & Drug Administration (FDA)-approved drugs. Notably, several validated hits were Wnt/β-catenin pathway activators and were consistent with previous screening in fibroblast cell lines (49). Other recent evidence supports the role of Wnt signaling in airway regeneration and aging (50, 51), suggesting that manipulation of Wnt pathway might represent an opportunity to develop proregenerative airway therapies (52). Further studies to characterize the impact of Wnt activators on Wnt and associated pathways may improve our ability to produce engineered airway basal cell therapies.

In summary, close collaboration between basic scientists, surgeons, and other clinicians is enabling more rapid progress in the translation of lung therapeutics. Benefits include improving the clinical relevance of fundamental research and improving preclinical model systems to reflect clinical experience.

### Session VI: Compassionate Use of Cell and Gene Therapie*s*

Although no approved CGTs for respiratory diseases and critical illnesses exist (53), several cell- and gene-based products are currently marketed or are at advanced stages of clinical testing for other various indications (https://www.cellandgene.com/doc/navigating-expanded-access-for-experimental-cell-gene-therapies-0001). Novel gene therapies are also increasingly approved but their high price tags raise questions of accessibility and equity. These developments have created a rapidly changing regulatory and business environment and have resulted in increased patient interest in nontrial access to experimental CGTs. This session featured presenters Dr. Alison Bateman-House (Research Assistant Professor at NYU Grossman School of Medicine), Prof. Patti Zettler (Professor, Ohio State University College of Law), and Dr. Dean Lee (Professor at Nationwide Children’s Hospital). This session was cosponsored by the International Society for Cell & Gene Therapy (ISCT) and provided insight into the complex clinical, bioethical, and regulatory issues surrounding “compassionate use” of CGTs, especially in a US context with FDA authorization via the “Expanded Access” pathway.

Dr. Patti Zettler, as the Chair of the ISCT’s Expanded Access Working Group, introduced the basics of the expanded access pathway and presented the current work and future directions of the Group. Since expanded access is a mechanism for nontrial use of unapproved interventions, including CGTs, it principally concerns patients with serious or life-threatening conditions with no alternative or satisfactory treatment. Therefore, the main purpose of this mechanism is to treat a condition rather than generate clinical data related to efficacy. The FDA has greatly improved and simplified the process for expanded access requests and authorizes their vast majority [e.g., in 2021 the FDA Center for Biologics Evaluation and Research (CBER) authorized 411 of 417 requests]. Although nontrial access to CGTs is also potentially available through Federal and State “Right-to-Try” laws, “right to try” is rarely used and it does not offer clear-cut benefits for patients relative to FDA’s expanded access. Given the increasing interest in expanded access for CGTs, the ISCT Expanded Access Working Group has published a report on the benefits, limitations, and emerging bioethical issues of this mechanism (54). Three salient issues identified are *1*) balancing desires for nontrial access to experimental CGTs versus need for evidence generation, *2*) the potential problematic use of expanded access as a route to premature commercialization, and *3*) equity in expanded access. Importantly, the Group is currently working toward developing a set of guidelines for expanded access stakeholders, such as CGT developers, clinicians, and patients.

The particularities of expanded access for gene-based therapies were highlighted by Dr. Bateman-House, who chairs the NYU Grossman School of Medicine Working Group on Compassionate Use & Preapproval Access (CUPA). There is a growing number of approved, potentially curative, gene therapies in the United States (https://www.fda.gov/vaccines-blood-biologics/cellular-gene-therapy-products/approved-cellular-and-gene-therapy-products). At the same time, premarket access to such therapies depends on the developer and there are instances in which such access has not been granted (https://cdn.pfizer.com/pfizercom/Policy-paper-Expanded-Access-to-Gene-Therapies-FINAL-2023.pdf). Several factors may affect expanded access to gene therapies. Several of these therapies concern rare diseases with small patient populations. As the manufacturing process for gene therapy vectors is expensive, complex, and not amenable to scale up, there is often less product available for expanded access. Another critical factor is the irreversibility of gene therapies that affects eligibility of patients for other types of treatment or future participation in clinical trials. Complex safety issues, such as long-term monitoring, possibility of virus shedding by gene therapy recipients, and preexisting antibodies to viral vectors may further complicate nontrial patient access to experimental gene therapies. Transparent and detailed expanded access policies for gene therapies are important to balance safety concerns, commercialization decisions, and bioethical considerations (55).

As forcefully argued by Dr. Lee, bioethical issues may become particularly salient in the case of compassionate use of cell-based therapies for vulnerable patient populations, such as pediatric patients. There is a dearth of clinical studies involving pediatric subjects, despite efforts to increase industry interest in pediatric indications such as the Orphan Drug Act (1986) and the FDA Pediatric Rule of 1998, which can lead to a vicious circle of limited data generation and overreliance on compassionate use therapies. The current drug development paradigm, where most stages beyond early development are taken over by industry, is not conducive to cell-based therapies for pediatric patients. If academic institutions are to address the unmet need for novel pediatric cell therapies, they need to fulfill the roles of inventor, manufacturer, sponsor, and clinician. Dr. Lee drew on his extensive experience with natural killer (NK) cell-based adoptive therapy to discuss barriers to successful academic deployment of cell therapies. The development of GMP facilities within academic medical institutions that will seamlessly move products from the preclinical and qualification stages to clinical manufacturing is critical. Challenges such as high cost, limited availability, and moderate-to-low efficacy might be addressed by allogeneic, universal donor manufacturing in some contexts. In addition, academic cell therapy programs, such as the Cellular Therapy and Cancer Immunology Program at Nationwide Children’s Hospital, require tight coordination and service integration between numerous departments, including legal, regulatory, financial, and clinical care. Pediatric-expanded access, especially in emergency requests, also greatly benefits from the effective establishment and maintenance of such programs, as shown by the emergency IND for and successful cytotoxic T cell (CTL) treatment of a 29-wk-old premature infant who presented with systemic adenovirus infection (56). Collectively, these three presentations and the lively discussion that followed highlighted the keen interest of basic and clinical researchers in the complex bioethical and regulatory issues linked to the compassionate use of CGTs.

### Session VII: Epithelial Plasticity: Cells Going Awry in Lung Injury

The importance of epithelial-mesenchymal interactions in lung development, homeostasis, and response to injury have become increasingly clear. This session focused on the plasticity of epithelial cell types in response to cross-talk signals from other niche components in health and disease. During normal repair of the lung, AT2 cells proliferate and differentiate into alveolar type 1 cells (AT1s) to repair the alveolar lining and restore normal structure and function. In both humans and murine models, injured or stressed alveolar epithelial cells (AECs) enter a transitional state in response to a variety of cues, exiting the cell cycle and adopting a transcriptional program distinct from AT2 or AT1 (57–59). Dr. Rachel Zemans (Professor, University of Michigan) discussed the dynamics of AT2/AEC transitional states/AT1 and the consequences of persistence of the Krt8^hi^ transitional state for lung injury and repair (60). The transcriptome of these Krt8^hi^ transitional cells is highly conserved across a variety of murine models of lung injury and fibrotic remodeling (61). Although Krt8 itself may not directly regulate AT1 differentiation, it appears to promote fibrosis through a complex set of interactions with other cell types, including the recruitment of profibrotic macrophages, which further maintain the transitional state through the elaboration of transforming growth factor-β (TGF-β) and IL-1β, and the activation of fibroblasts.

Within the distal lung, transcriptional profiling has identified three functionally distinct populations of platelet-derived growth factor receptor alpha (PDGFRA)^+^ fibroblasts, which are regulated by different signals during both development and in response to injury: lipofibroblasts, myofibroblasts, and matrix fibroblasts (62). Dr. Anne-Karina Perl (Professor at Cincinnati Children’s Hospital Medical Center) elucidated several important ways in which the mesenchyme instructs epithelial differentiation. During hyperoxia, the total number of PDGFRA^+^ cells is reduced, with the remaining PDGFRA^+^ fibroblasts taking on a lipofibroblast function while losing myo- and matrix-fibroblast function. Functionally, fibroblasts from hyperoxia-treated mice fail to support epithelial differentiation in alveolar organoids (63). Furthermore, idiopathic pulmonary fibrosis (IPF), a disease of the elderly, is similarly characterized by both impaired AT1 differentiation and loss of interstitial matrix fibroblasts. The role of aging in these events was evaluated in reciprocal organoid experiments. Aged epithelial cells retain their ability to differentiate when combined with young fibroblasts, whereas aged fibroblasts lose the ability to promote epithelial differentiation. Activation of PDGFA signaling supports a transition from myofibroblasts to matrix fibroblasts and the subsequent conversion of AT2s to AT1s, which is necessary for regeneration and repair (64). Dr. Jaymin Kathiriya (Assistant Professor at Mt. Sinai) discussed additional ways in which in vivo plasticity of human AECs (hAEC) depends on mesenchymal input. Coculture of hAT2s with adult human lung mesenchyme (AHLM) rich in PDGFRA^+^ fibroblast populations results in their transdifferentiation to KRT5^+^ basal cells, recapitulating events in IPF, in which AEC2s are lost and KRT5^+^ metaplastic alveolar basal cells appear. Trajectory analyses demonstrate that hAT2s pass through two distinct alveolar-basal intermediates (ABI1 and AI2), which share features of transitional-state cells described by Dr. Zemans and others (60, 65, 66). Cultured AHLM and IPF mesenchyme shared several features, including expression of CTHRC1, TGF-β1, and bone morphogenetic protein (BMP) antagonists. This aberrant, profibrotic mesenchyme promotes basal cell transdifferentiation and is spatially associated with ABIs in human IPF lungs. Thus, ABIs derived from hAT2s recapitulate the aberrant basaloid cells present in human IPF lungs, and the transition of hAT2s to basal cells is driven by the presence of a profibrotic niche formed by adjacent mesenchymal cell populations (67).

Finally, the effects of the mechanical environment on the plasticity of cells were explored. Using a 2-D model of weighted-agarose compression, Akash Gupta (MD/PhD Candidate, University of Groningen), an Outstanding Abstract award winner, demonstrated that contact-compression of human bronchial epithelial cells (HBECs) induced both proinflammatory and profibrotic responses. In summary, this session delved into the complex interactions between cell types, matrix, and mechanical forces occurring in normal lung development, homeostasis, and cellular responses to environmental perturbations and injury.

### Session VIII: Dame Julia Polak Memorial Bioengineering Session: Mechanotransduction in Lung Development and Disease

The lung is a mechanically active organ whose functions and structures are strongly influenced by mechanical forces during normal homeostasis, disease progression, and embryonic development (68). In this session, we discussed the role of mechanical forces with a focus on the developing lung. During the session, speakers introduced multiple innovative bioengineering approaches used to determine the links between mechanical forces and embryonic development. As the lung develops, the epithelium undergoes both morphogenesis and regional specification in the form of differentiation. It is often challenging to uncouple morphogenesis from differentiation, and it remains unclear whether one is upstream of the other. Dr. Celeste Nelson (Professor, Princeton University) discussed bioengineering approaches to restore branched morphology to mutant mouse lungs that display disrupted embryonic lung development. Surprisingly, in *Yap*/*Taz* knockout mice, the patterns of normal branching morphogenesis and differentiation were restored when the epithelium was placed under physical constraints that mimic those imposed by the surrounding airway smooth muscle layer. These findings suggest that at least one of the epithelial cell-fate decisions in the early embryonic lung is regulated by mechanical force as a downstream process of morphogenesis. Using embryonic lungs cultured ex vivo, Dr. Victor Varner (Associate Professor, University of Texas) studied epithelial buckling as a key step in airway branching morphogenesis (69). In the developing lung, epithelial buckling morphogenesis and the formation of multiple new supernumerary buds could be induced by an ectopic source of FGF-10 in the pulmonary mesenchyme. They further tested the role of mechanical properties of pulmonary mesenchyme in epithelial buckling. Using a photo-cross-linking approach, they locally modified the mechanical properties of the developing pulmonary ECM, without perturbing endogenous patterns of proliferation and apoptosis. As the pulmonary mesenchyme became locally stiffer, epithelial buckling was suppressed, and FGF-10-induced budding morphogenesis was disrupted. Together, the first two presentations in this session highlighted how a better understanding of branching morphogenesis and its relationship with the local mechanical environment might be used to direct tissue morphogenesis in support of lung regeneration or engineered tissue replacements. Dr. Yekaterina Miroshnikova (Tenure track investigator at the NIH) next discussed how mechanical force remodels nuclear architecture, chromatin state, and global gene expression patterns in both somatic and embryonic stem cells (70). In studies using quantitative imaging and sequencing approaches, they discovered a mechanism by which nuclear deformation triggers changes in chromatin architecture and gene expression, resulting in epigenetic memory affecting cellular lineage progression. While only beginning to be investigated in the context of lung development, such nuclear mechanotransduction mechanisms might serve as important links from the tissue-level mechanical environment to cell fate, with implications for understanding how lung cell fates emerge in development or are disturbed in disease. Finally, Nika Gvazava (PhD Candidate, Lund University), an Outstanding Abstract awardee, introduced the application of label-free high-resolution optical photothermal midinfrared (O-PTIR) spectroscopy to study the tissue molecular composition. In precision-cut lung slices, they shared how label-free O-PTIR can be used on living 3-D tissue to assess diverse biological molecules at subcellular resolution. The collection of methods shared in this session should pave the way toward a better understanding of how the mechanical environment influences lung development and might be harnessed to support lung repair.

### Session IX: Challenges and Controversies in Human Mesenchymal Stromal Cell Therapy

Mesenchymal stromal cells (MSCs) were initially identified in bone marrow as fibroblastic colony-forming units and thought to provide a supportive stroma for hematopoietic stem cell maturation. They act as a stem cell with differentiation capacity to generate mesodermal lineages, bone, fat, and cartilage. MSCs have been isolated from adipose, placental, and other tissues and have become increasingly noted for their paracrine properties secreting a range of anti-inflammatory mediators, growth factors, and other substances (71–75). There is a wealth of preclinical data in animal models of lung injuries, demonstrating the efficacy of both systemic and direct airway MSC administration. However, despite several clinical investigations in pulmonary diseases and critical illnesses, there is no clear pattern of efficacy. Initial studies highlight MSC anti-inflammatory and regenerative capabilities, suggesting the potential for therapeutic benefit in ARDS, sepsis, COPD, pulmonary fibrosis, and asthma (71–74). However, controversies arise from inconsistent results in clinical trials, raising questions about efficacy and optimal protocols. Some studies report positive outcomes, whereas others indicate limited benefits. In addition, concerns about precise mechanisms of action of MSCs, how in vitro testing/potency correlates to in vivo activity, the potential for adverse effects, long-term safety, and ethical considerations persist. Although the progress of MSC translation into the clinical arena is slower than it was initially hoped, the field continues to work tirelessly toward the goal of widespread therapeutic use of MSCs. In this session, Dr. Jaques Galipeau (Professor, University of Wisconsin and President of the ISCT), Dr. Lauralyn McIntyre (Associate Professor, University of Ottawa, Canada), and Dr. Tracy Heng (Associate Professor, Monash University, Australia) presented ongoing research that addresses these controversies to determine the true potential and risks of MSC therapy for lung diseases.

One reason for controversial results in the administration of MSC in clinical trials is the incomplete understanding of their fate after delivery. Several studies have demonstrated that in vivo MSCs undergo apoptosis and subsequent clearance by phagocytes and this results in the anti-inflammatory reprogramming of the immune response. Previously, using apoptosis-refractory MSCs, Dr. Heng demonstrated that MSC apoptosis and subsequent efferocytosis by alveolar macrophages are required for their full immunosuppressive effects in the mouse model of ovalbumin-induced asthma (76). Here, Dr. Heng presented new data on the role of MSC apoptosis in their anti-inflammatory actions using an LPS-induced lung injury model and demonstrated a key role of red pulp macrophages in the spleen for clearance of the apoptotic MSCs, as the removal of spleen abolished the therapeutic benefit of MSCs. These findings provide new insights into the mechanisms of action of MSC cell therapies and potentially infers the efferocytic clearance as a potency assay for MSC efficacy.

Dr. Galipeau summarized the MSC trials conducted to date and pointed out that despite the overwhelming number of published preclinical studies that have demonstrated therapeutic benefit with MSC treatment, there have only been a handful of MSC products that have achieved regulatory approval status (first MSC market approval was Alofisel, granted in March 2018). Concerns with regard to the postthaw viability of cryopreserved MSC products were raised, given there is increasing trends of using thawed MSC products in industry-sponsored clinical investigations (77). Using a preclinical mouse model of colitis, he presented data comparing fresh, thawed, and dead MSCs and showed that the use of thawed MSC products may have impaired “fitness” and less ability to persist in animals compared with fresh products. Finally, he discussed an upcoming phase 1 clinical trial that will evaluate the safety and tolerability of direct salivary gland injection of autologous, fresh, IFN-γ-activated bone marrow-derived MSCs for the treatment of radiation-induced xerostomia [Marrow-Derived Autologous Stromal Cells for the Restoration of Salivary Hypofunction (MARSH)]. This is a dose-escalation trial with a 3 + 3 design, with a plan to recruit up to 30 patients.

Dr. McIntyre presented the multicenter, double-blind phase II clinical trial design of Umbilical MSCs as Cellular Immunotherapy for Septic Shock (UC-CISS II). The phase I CISS trial established that MSCs were safe and that a randomized controlled trial (RCT) is feasible. Based on these data, the investigators have planned a phase II RCT (UC-CISS II) at several Canadian academic centers. This trial will assess measures of clinical efficacy (primary outcome), as well as biomarkers, safety, clinical outcome measures, and a health economic analysis (secondary outcomes). UC-CISS II will randomize 296 ICU-admitted patients with septic shock, who will either receive 300 million cryopreserved, allogeneic, umbilical cord-derived MSCs or placebo. Collectively, this session demonstrated the need for further investigation of the mechanisms mediating the MSC effects in the injured lungs, the use of physiologically relevant models that can better model human disease, and finally, the improvement of MSC clinical trial design to gain a better understanding of the ideal patient populations who may benefit most from receiving MSC therapy.

### Session X: The Matrix Revealed: Understanding Extracellular Matrix Influence of Repair and Regeneration

The ECM guides cell fate through a variety of different biochemical and mechanical cues. This session highlighted the importance of including physiologically relevant stimuli, like mechanical stretch and stiffness, into models aiming to replicate the in vivo conditions of lung tissue. Leveraging engineering techniques to systematically investigate the role of specific changes to the ECM will improve our understanding of cell signaling pathways and mechanisms involved in disease and repair and may lead to greater likelihood of successful therapeutic development (68). Leigh-Ann Antczak (PhD Candidate, Virginia Commonwealth University) was the Outstanding Abstract achievement award presenter in this session and spoke about dual-stiffness decellularized ECM hydrogels or hybrid gels. ECM derived from healthy or COPD human lungs was integrated into the hydrogel and supported cell viability. Comprehensive biological and material characterizations of these constructs revealed similar matrix stiffness, pore size, and elastin-to-collagen ratios to that of native lung tissues. This emerging ex vivo technique also has the potential to be adapted to other organ systems. Similarly in Dr. Harikrishnan Parameswaran’s (Associate Professor, Northeastern University) work, dual-stiffness materials were used to assess the intercellular communication alterations due to matrix stiffness (78). After a series of experiments, diverging results appeared. Stiffening had negligible impact on agonist-induced calcium frequency in sparsely seeded airway smooth muscle cells (ASMCs) but evoked different calcium responses that were dependent on matrix stiffness when cultures were confluent. Follow-up studies applied shear stresses to ASMCs and evaluated how membrane stretch from intercellular forces influenced the same pathway that is involved in membrane lipid phosphoinositide 4,5-bisphosphate (PIP_2_) hydrolysis. Interestingly, ASMCs were unable to maintain tension when this phospholipase C (PLC) activation pathway was inhibited. Together, these results suggest that force generation and maintenance in the ASMC is a collective phenomenon that results from cell-cell and cell-matrix interactions.

Dr. Amanda Tatler’s (Associate Professor, University of Nottingham) work also built on the concept of using stretch studies. In the asthmatic lung, transforming growth factor-β (TGF-β) is a key mediator of tissue remodeling that can be activated through increased matrix stiffness or stretch. The ECM in the asthmatic lung is altered compared with non-asthmatic lung (79), with a greater degree of cross linking due to altered lysyl oxidase family members in the asthmatic tissues (80). It was hypothesized that ECM alterations might also contribute to TGF-β activation in asthmatic ASMCs (81). To test this theory, asthmatic and nonasthmatic cells were placed on matrices derived from asthmatic or nonasthmatic lung tissue. TGF-β activity decreased when the asthmatic cells were cultured on the opposite matrix (e.g., nonasthmatic matrix) and implicates ECM cross linking as another possible mechanism of TGF-β activation. By inhibiting lysyl oxidase-like 2 (LOXL2), it was possible to decrease TGF-β activation. These findings suggest that therapeutic benefits may come from targeting LOXL2 or other lysyl oxidases (82, 83). Finally, Dr. Núria Gavara (Assistant Professor, University of Barcelona) focused on the biomechanical environment in lung tissues and the importance of considering these factors when modeling lung diseases. She discussed atomic force microscopy (AFM), highlighting the strengths of this technique (e.g., ability to measure extremely soft substrates, high resolution across multiple scales, and visual options to correlate stiffness and topography). It is important to note that sample processing can significantly alter any mechanical property measurements and often differs significantly across various platforms and institutions (84). These concepts were exemplified in her in situ decellularization experiments that aimed to preserve native tissue features, such as inflation volume, for mechanical testing (85). Surprisingly, the study showed that *1*) stretching fibrotic lung scaffolds softens the tissue and *2*) strain stiffening responses differed depending on whether the applied stretch was above or below a 5-kPa threshold, a value that often falls in the range of typical behavioral crossovers. Depth sensing by cells and increased sensitivity on soft substrates often contribute to this occurrence (86). Focusing future efforts on developing hydrogels and model systems that dynamically soften or exhibit stiffness in the lower pascal range may provide core insights about repair and disease progression.

### Session XI: Setting Priorities and Recommendations Regarding Funding Future Research

In the final session, participants took part in an active discussion designed to get input on the limitations, challenges, and needs essential for continued progress in basic and translational research relating to the advancement of the field of lung stem cells and bioengineering. The broad field of lung regenerative medicine and engineering continues to evolve at an accelerating and exciting pace. The NIH, National Science Foundation (NSF), nonprofit respiratory disease foundations, and other sources of scientific and funding support remain positive and continue to be crucial for continued development. As in past conference reports, a series of scientific and funding recommendations resulting from discussions at the conference and postconference surveys are presented in Table 1. These recommendations continue to evolve and reflect a growing interest in the application of bioengineering approaches and technologies, including recent advances in “omics,” organoids, and analytical technologies, to the study of lung biology and diseases. This final session used live polling to assimilate anonymous responses from participants of the conference to guide discussion and craft overall recommendations; these addressed key theme areas of the conference, including challenges in engineering new models of lung disease and repair, a topic of increasing importance in understanding intercellular interactions, and the impact of cellular niches on furthering our understanding of lung biology and the feasibility of successful cellular therapies.

**Table 1.** Conference summary, recommendations, and focus areas

*Basic Science: Analysis and Visualization of Cellular Plasticity*
Continue progress in applying a system-level approach to discover interactions through mining of single-cell omics data to identify fundamental cell signaling pathways and networks in homeostasis and disease
Increase the development and accessibility to platforms for data integration, analysis, and functional annotation across different omic modalities
*Basic Science: Complex Cell: Niche Interactions in Homeostasis and Disease*
Investigations to understand the mobilization endogenous cells are exciting current approaches for repairing damaged airway and alveolar epithelium
Increased understanding of the complexity of the regulation of lung stem and progenitor cells by their direct, in vivo, niche is vital to the success of any mechanism for restoration of homeostasis in a diseased lung
Understand the influence of immune cells on airway regeneration and the potential for immune cell targeting to coordinate lung repair and regeneration
*Basic Science: Bioengineering and Mechanotransduction*
Continue to explore lung tissue bioengineering approaches such as artificial matrices, 3-D culture systems (e.g., extracellular matrix environments for organoid culture), 3-D bioprinting, and other novel approaches for generating lung ex vivo and in vivo from stem cells, including systems that facilitate vascular development
Define the consensus endpoints for functional evaluation and validation of engineered lung tissue
Understand the parameters of extracellular matrix that define its regulation of airway epithelial function in health and disease
*Translational Science: Cell Therapy—Advancing Cellular Therapeutics*
Invest in the development of more physiologically relevant and human-engineered models to accelerate our ability to understand both lung disease pathogenesis and injury repair mechanisms
Integrate lung stem cell science into multidisciplinary teams (e.g., with clinical, surgical, regulatory, informatics input, etc.)

3-D, three-dimensional.

In brief, the polling indicated several challenges that limit the ability to apply novel materials and models to fundamental, basic scientific questions regarding airway regeneration and lung biology. These ranged from challenges in finding suitable funding mechanisms and collaborators with the relevant expertise to a lack of knowledge on the design criteria to replicate lung tissues. Many laboratories are now using or plan to use single-cell proteomics and transcriptomics; however, access to spatial technologies appears to be a limiting factor in pursuing this technology. Unsurprisingly, the cost of using/accessing advanced technologies is the biggest barrier to adopting new techniques. Suggestions to increase accessibility center around access to centralized facilities and supplemental funding options from the core funding bodies. In addition, access to expertise and availability of training were challenges that could be addressed through creation of a centralized resource with training courses. Several suggestions were made to increase interactions with biomedical engineers and cell biologists, including options for regular online meetings, funding agency-led workshops, funding mechanisms to support sabbaticals, and online chat forums.

## CONCLUSIONS

The meeting demonstrated our ongoing and substantial improvement in the understanding of lung stem/progenitor cell biology, advancements in the use of new model systems, and innovative approaches to engineering lung tissue over recent years. The integration of omics technologies into lung regeneration workflows continues to accelerate both our basic understanding and the translational potential of findings in the field. By convening active researchers from multiple disciplines, clinicians, policymakers, and industry scientists, the conference provides a platform for cross fertilization of ideas. The conference continues to focus on the promotion of junior scientists to stimulate innovation and retain talent within the lung bioengineering field. The discussion at the conference, the resulting recommendations, and the results of the participant survey should guide the next phase of lung bioengineering research as the field seeks strategies to provide functional lung tissue for patients with lung diseases.

## GRANTS

This work was supported by National Heart, Lung, and Blood Institute Grant R13 HL170688-01 and National Science Foundation Grant 2327935.

## DISCLOSURES

A.L.R. and D.J.W. received funding from National Heart, Lung, and Blood Institute R13 Conference Grant and the National Science Foundation to support the conference. None of the other authors has any conflicts of interest, financial or otherwise, to disclose.

## AUTHOR CONTRIBUTIONS

R.E.H., C.M.M., L.I., Y.A., M.F.B., J.K.B., R.L.H., P.S.H., A.D.K., S.H.J.M., A.V.M., J.-A.P., S.D.R., D.J.T., A.E.T., S.V., C.M.W., P.J.Z., D.J.W., and A.L.R. drafted manuscript; R.E.H., C.M.M., L.I., Y.A., M.F.B., J.K.B., R.L.H., P.S.H., A.D.K., S.H.J.M., A.V.M., J.-A.P., S.D.R., D.J.T., A.E.T., S.V., C.M.W., P.J.Z., D.J.W., and A.L.R. edited and revised manuscript; R.E.H., D.J.W., and A.L.R. approved final version of manuscript.
